# Feasibility study of computed tomography texture analysis for evaluation of canine primary adrenal gland tumors

**DOI:** 10.3389/fvets.2023.1126165

**Published:** 2023-08-30

**Authors:** Kyungsoo Lee, Jinhyong Goh, Jaeyoung Jang, Jeongyeon Hwang, Jungmin Kwak, Jaehwan Kim, Kidong Eom

**Affiliations:** ^1^Department of Veterinary Medical Imaging, College of Veterinary Medicine, Konkuk University, Seoul, Republic of Korea; ^2^Jang Jae Young Veterinary Surgery Center, Seong-nam, Gyunggi-do, Republic of Korea; ^3^Helix Animal Medical Center, Seoul, Republic of Korea; ^4^Saram and Animal Medical Center, Yongin-si, Gyunggi-do, Republic of Korea

**Keywords:** computed tomography, texture analysis, adrenal gland tumor, adenoma, adenocarcinoma, pheochromocytoma, canine

## Abstract

**Objective:**

This study aimed to investigate the feasibility of computed tomography (CT) texture analysis for distinguishing canine adrenal gland tumors and its usefulness in clinical decision-making.

**Materials and methods:**

The medical records of 25 dogs with primary adrenal masses who underwent contrast CT and a histopathological examination were retrospectively reviewed, of which 12 had adenomas (AAs), 7 had adenocarcinomas (ACCs), and 6 had pheochromocytomas (PHEOs). Conventional CT evaluation of each adrenal gland tumor included the mean, maximum, and minimum attenuation values in Hounsfield units (HU), heterogeneity of the tumor parenchyma, and contrast enhancement (type, pattern, and degree), respectively, in each phase. In CT texture analysis, precontrast and delayed-phase images of 18 adrenal gland tumors, which could be applied for ComBat harmonization were used, and 93 radiomic features (18 first-order and 75 second-order statistics) were extracted. Then, ComBat harmonization was applied to compensate for the batch effect created by the different CT protocols. The area under the receiver operating characteristic curve (AUC) for each significant feature was used to evaluate the diagnostic performance of CT texture analysis.

**Results:**

Among the conventional features, PHEO showed significantly higher mean and maximum precontrast HU values than ACC (*p* < 0.05). Eight second-order features on the precontrast images showed significant differences between the adrenal gland tumors (*p* < 0.05). However, none of them were significantly different between AA and PHEO, or between precontrast images and delayed-phase images. This result indicates that ACC exhibited more heterogeneous and complex textures and more variable intensities with lower gray-level values than AA and PHEO. The correlation, maximal correlation coefficient, and gray level non-uniformity normalized were significantly different between AA and ACC, and between ACC and PHEO. These features showed high AUCs in discriminating ACC and PHEO, which were comparable or higher than the precontrast mean and maximum HU (AUC = 0.865 and 0.860, respectively).

**Conclusion:**

Canine primary adrenal gland tumor differentiation can be achieved with CT texture analysis on precontrast images and may have a potential role in clinical decision-making. Further prospective studies with larger populations and cross-validation are warranted.

## 1. Introduction

Canine primary adrenal gland tumors are rarely reported, accounting for 0.17–0.76% of all tumors in dogs (1). Adrenal gland tumors can be classified as having a cortical or medullary origin, being benign or malignant, and functional or non-functional. Adenoma (AA) and adenocarcinoma (ACC) that arise from the cortex are the most common, accounting for 75% of all canine adrenal gland tumors. Pheochromocytoma (PHEO), which originates from the medulla, is the next most common (1–5). Adrenalectomy is indicated when an adrenal mass is suspected to be malignant; therefore, preoperative diagnosis through diagnostic imaging findings, clinical signs, and blood test results including cortisol, plasma, or urine metanephrine levels is important. Adrenal masses can exhibit specific symptoms depending on the pathology, but non-functional adrenal masses, also called “incidentalomas,” are frequently recognized on routine abdominal imaging in older dogs (6). In such cases, the diagnosis is not straightforward, and computed tomography (CT) may play an important role in differentiating adrenal gland tumor types.

CT is commonly used to evaluate canine adrenal gland tumors, including their shape, parenchymal heterogeneity, and vascular invasion. A study of 17 dogs with primary adrenal gland tumors reported a significant correlation between the histological diagnosis and CT features; however, overlapping characteristics between tumor types were identified, indicating that CT alone has limitations in distinguishing adrenal gland tumors (7). Another study of 36 dogs with adrenal gland tumors showed that differential diagnosis is possible through percentage enhancement washout, relative percentage washout, and enhancement washin and washout, which can be obtained using triple-phase CT (8). In addition, morphological features and precontrast attenuation values provide useful information. ACC showed lower minimum and maximum Hounsfield unit (HU) values than AA or PHEO in the precontrast images, and PHEO tended to show a more lobulated shape than other tumors (8). Another previous study presented a cut-off value of the precontrast Hounsfield unit (HU) value and size of the contralateral normal adrenal gland in differentiating ACC from PHEO (9). ACC tended to show a precontrast HU lower than 39, whereas that value for PHEO was higher than 49. In addition, PHEO tended to have a contralateral normal adrenal size >6 mm.

Texture analysis is an emerging technique that can quantitatively and objectively evaluate tumor heterogeneity by analyzing the distribution and relationship between pixels or voxel gray levels on medical imaging (10). It has shown potential clinical value in the preoperative non-invasive diagnosis of tumors and staging, evaluation of the response to treatment, and assessment of outcome in human medicine (11–14). Texture analysis evaluates morphological features, first-order statistics, second-order statistics, and higher-order statistics. Morphological features describe the geometric properties of the lesion, such as the volume, diameter, and surface area. First-order statistics describe the distribution of voxel values, regardless of their spatial relationships. These features are also known as intensity-based features that quantify the overall tumor intensity or density (such as mean and median), spread, and shape of the distribution (such as variance, interquartile range, skewness, and kurtosis). Second-order statistics, also known as texture features, describe the spatial relationship between adjacent voxels, such as the gray-level co-occurrence matrix (GLCM), gray-level run-length matrix (GLRLM), gray-level dependence matrix (GLDM), and gray-level size-zone matrix (GLSZM) (15, 16).

In human medicine, an adrenal incidentaloma is an incidentally identified adrenal lesion that is >1 cm (17). A list of differential diagnoses includes non-functioning AA, hyperfunctioning AA, metastasis, and primary adrenal gland tumors, such as PHEO and ACC. A definitive diagnosis is important because the treatment can vary from observation to resection. Various methods have been proposed for a more accurate diagnosis, including multiphasic CT with contrast enhancement, adrenal biopsy, nuclear or functional imaging (e.g., 18F-fluorodeoxyglucose positron emission tomography and magnetic resonance imaging chemical shift), and spectrometry for measuring the steroid precursor (17, 18). However, several limitations have been identified with these methods, and CT texture analysis has been suggested as a promising method for differentiating malignant from benign adrenal gland tumors (19).

Texture analysis has been introduced in several veterinary studies. CT texture analysis has been used to evaluate pulmonary parenchyma in dogs with pulmonary thromboembolism, to identify prognostic factors in dogs with primary lung tumors, and to differentiate between benign and malignant hepatic masses (20–22). Other studies have shown the feasibility of texture analysis using MRI for identifying meningiomas and muscular dystrophy (23–25). However, this novel diagnostic imaging approach has not yet been used for distinguishing primary adrenal gland masses. Given the possibility of additional non-invasive diagnosis in conventional imaging analysis of human adrenal gland tumors, there is good reason to investigate the feasibility of CT texture analysis in dogs with adrenal gland tumors. Therefore, the present study aimed to characterize the texture features of canine primary adrenal gland tumors, including cortical AAs, ACCs, and PHEOs, using conventional CT evaluation. In addition, we aimed to assess the diagnostic performance of CT texture features using the receiver operating characteristic (ROC) curve and compare it with that of conventional CT evaluation.

## 2. Materials and methods

### 2.1. Ethics statements

Written informed consent was obtained from the owners for their participation in this study.

### 2.2. Animals and study design

#### 2.2.1. Case selection

CT datasets and medical records of dogs with adrenal masses measuring >1 cm in maximal diameter were retrospectively reviewed from four different institutions: Konkuk University Veterinary Medical Teaching Hospital, Jang Jae Young Veterinary Surgery Center, Helix Animal Medical Center, and Saram And Animal Medical Center. Only dogs with a preoperative contrast-enhanced CT scan and a definitive diagnosis based on histopathological examination findings of the adrenal mass after surgical removal were included. The following cases were excluded: those that did not undergo surgical removal and histopathological diagnosis; and those with benign adrenal lesions such as a lipoma, nodular hyperplasia, extramedullary hematopoiesis, loss of CT datasets, and ruptured adrenal mass.

#### 2.2.2. CT image acquisition

CT images were acquired from various multi-detector CT scanners using standardized abdominal imaging protocols. The scanner model and its manufacturer, slice thickness, tube voltage (kVP), and mAs are summarized in Supplementary Table 1. Post-contrast images were obtained using the bolus tracking technique or at a specific time after the contrast medium was injected (arterial phase, ~20 s; portal phase, ~40 s; delayed phase, 70 s to 2 min after the start of injecting the contrast medium). Because of the retrospective nature of this study, detailed protocols, including the manufacturer of the contrast medium and its dosage, injection rate, and detailed scanner parameters (e.g., pitch, rotation time, and reconstruction thickness), were not available.

### 2.3. Conventional CT analysis

#### 2.3.1. Quantitative features

**Figure 1:**
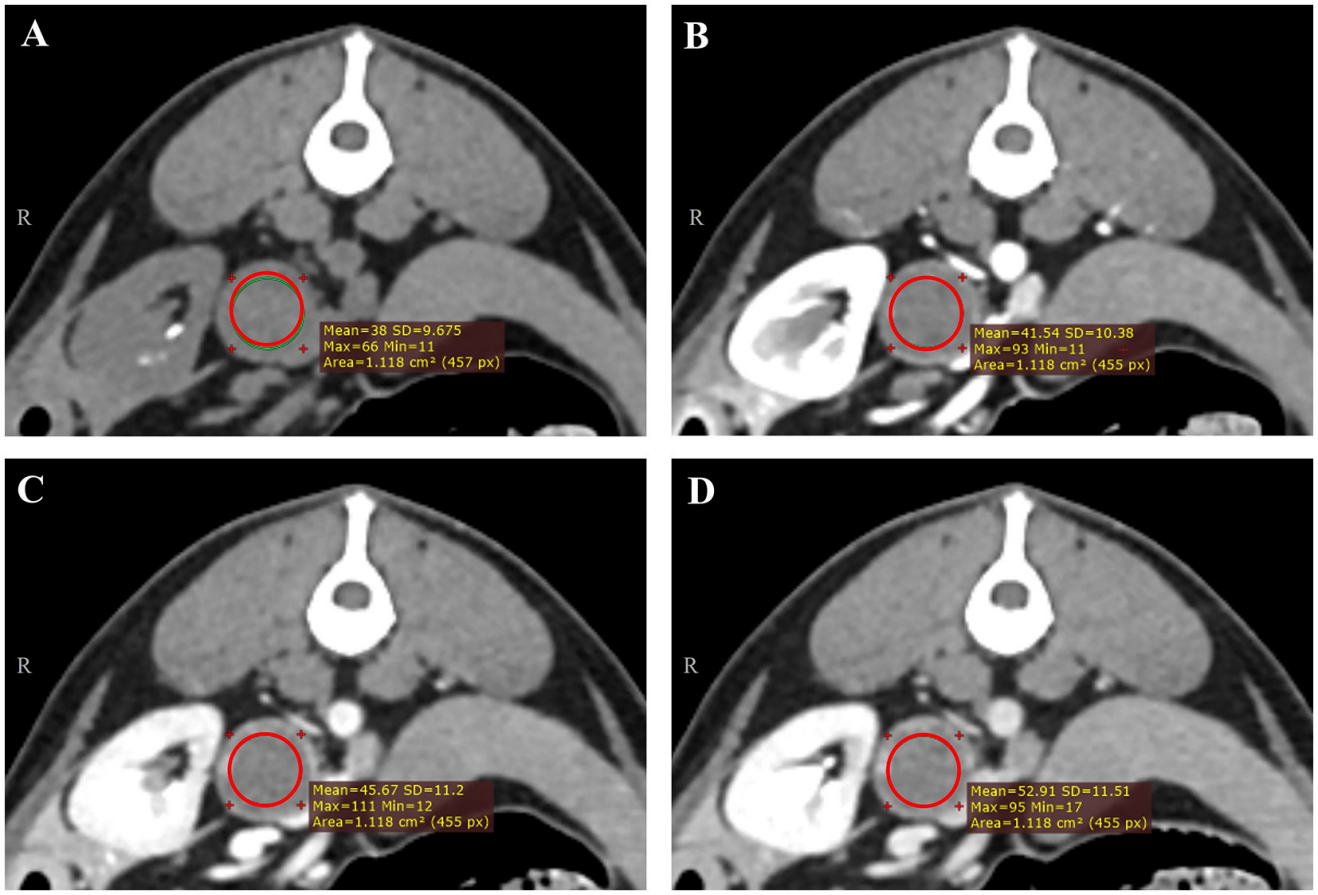
Representative triple-phase computed tomography images and measurement of the Hounsfield unit value of a canine adrenal gland tumor on precontrast **(A)**, arterial-phase **(B)**, portal-phase **(C)**, and delayed-phase images **(D)**. The region of interest (ROI) was manually drawn in the transverse plane of the maximal diameter, occupying at least two-thirds of the mass, except intra-tumoral calcification and the macroscopic fat area (< 0 HU). Each ROI was placed in the same area.

All CT images were reviewed retrospectively by a single investigator (Resident in diagnostic imaging of the Veterinary Medical Imaging Department of the Teaching Hospital of Konkuk University) using commercially available Digital Imaging and Communications in Medicine viewing software (Radiant™, Medixant, Poznan, Poland) over 2-week intervals, and a professor in the Veterinary Medical Imaging Department (KE) supervised the resident. The evaluation was performed without prior knowledge of the histopathological diagnosis. The maximal diameter on the short and long axes was measured in the transverse, sagittal, and dorsal planes. The mean, maximum, and minimum attenuation values of the mass were recorded in HU (Humean, HUmax, and Humin, respectively). Circular or ovoid regions of interest (ROIs) were manually drawn on the transverse plane of maximal diameter, occupying at least two-thirds of the mass (26). Intratumoral calcification and the macroscopic fat area (< 0 HU) were excluded. Each ROI was placed in the same area on the pre-contrast and post-contrast (arterial-, portal-, and delayed-phase) images (Figures 1A–D). Additionally, the difference in the mean attenuation value on pre-contrast images was calculated on each post-contrast (arterial-, portal-, and delayed-phase) image.

#### 2.3.2. Qualitative features

The following qualitative CT features were evaluated: the location of the lesion (right or left), shape of the mass (round, oval, and lobulated), mass contour (smooth and irregular), type of contrast enhancement in each phase (homogeneous and heterogeneous), pattern of contrast enhancement (stable, progressive, and washout), degree of contrast enhancement (no enhancement, minimal, mild, moderate, and intense), presence of rim enhancement, intratumoral calcification, and adhesion or invasion of adjacent vessels. The pattern of contrast enhancement was categorized as described in a previous study (27). A stable enhancement pattern was defined as minimal contrast enhancement of the lesion and constant attenuation values from the arterial phase to the delayed phase. A progressive enhancement pattern was defined as the gradual increase in the attenuation value of the lesion over time that reached its maximum in the delayed phase. The washout pattern was defined as peak contrast enhancement during the arterial phase, followed by a reduction in the attenuation value in the delayed phase. The degree of contrast enhancement was evaluated through the difference in mean HU between pre-contrast in the arterial phase, portal phase, and delayed phase, respectively: minimal (< 50 HU), moderate (≥50 and < 100 HU), and intense (≥100 HU). A previously reported CT grading system with a 7-point scale was used and modified to evaluate the possibility of adhesion or invasion of adjacent vessels (9). Grades 1 and 2 were considered to have no contact with adjacent vessels or a weak possibility of invasion or adhesion. Grades 3, 4, and 5 were considered moderate, and grades 6 and 7 showed compression of the vessel by the tumor or clear invasion of the vessel, which was categorized as a strong possibility.

### 2.4. Lesion segmentation and texture analysis

Texture analysis was performed using pre-contrast and delayed-phase images of adrenal lesions using the freely available open-source software 3D Slicer (version 5.1.0; https://www.slicer.org/). Eighteen of 25 dogs were included in the texture analysis; seven dogs were excluded because they were all imaged with different CT scanners and protocols and ComBat harmonization techniques could not be applied. The ComBat harmonization method is one of the most widely used techniques recently; it can compensate for the batch effect generated by different CT scanners and protocols (28, 29). In a previous study, only 18% of 89 radiomic features showed reproducibility when using six different image settings (30). This interscanner variability implies that the repeatability of radiomic features depends on the consistency of image acquisition and reconstruction. This technique effectively removes the batch effect while preserving the texture characteristics between multicenter radiomic studies (31).

**Figure 2:**
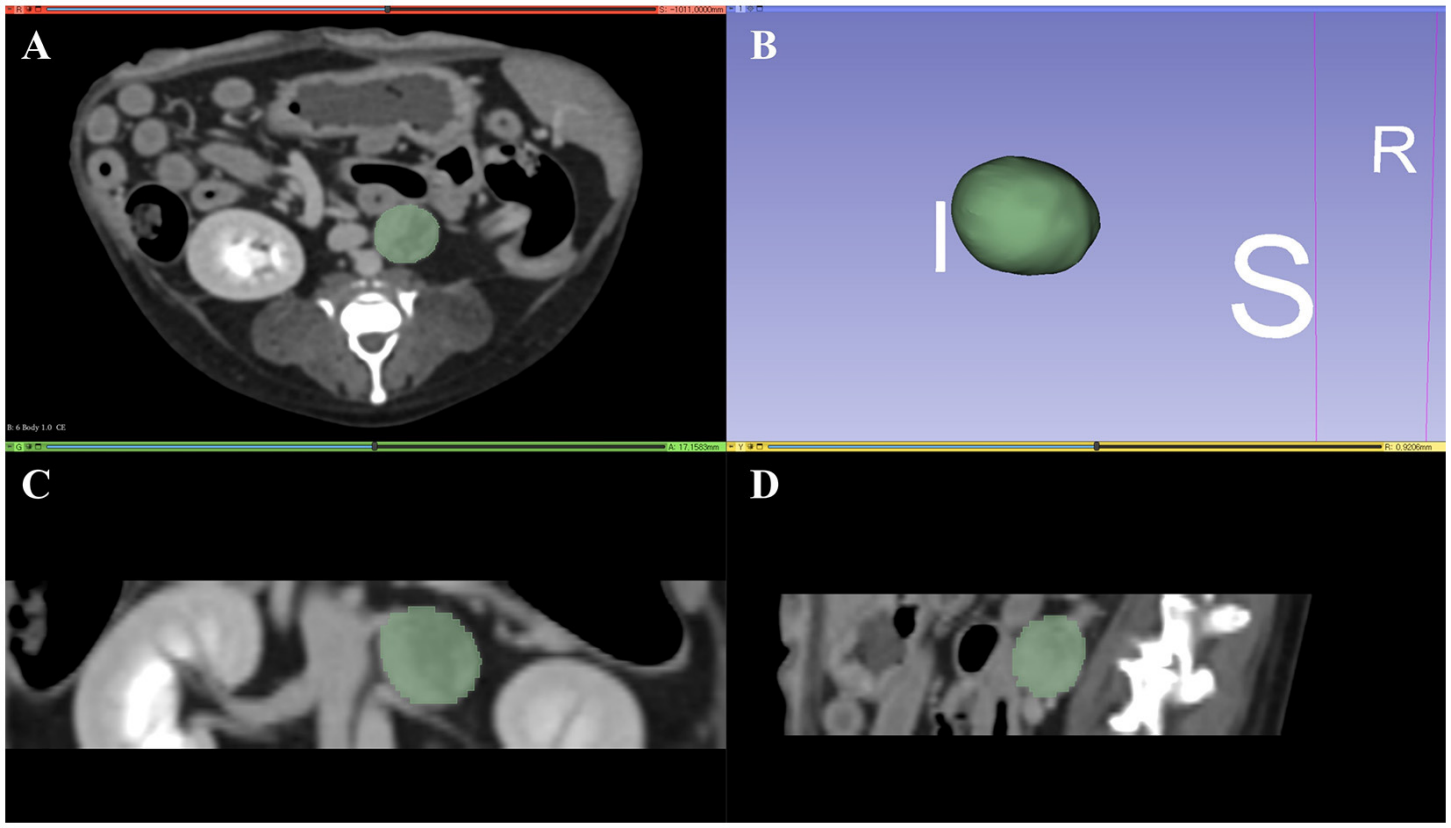
Representative tumor segmentation using 3D Slicer in the transverse plane **(A)**, dorsal plane **(C)**, and sagittal plane **(D)**. Three-dimensional reconstructed image of the tumor is represented in **(B)**. I, inferior; S, superior; R, right.

Semi-automatic segmentation was performed by drawing an ROI along the margins of the adrenal lesions (Figures 2A–D). After a volume of interest was created for each lesion, 18 first-order and 75 second-order statistics (24 GLCMs, 14 GLDMs, 16 GLRLMs, 16 GLSZMs, and 5 NGTDMs) were extracted using a 3D Slicer radiomic extension pack. The bin width was fixed at 25 HU to discretize the voxel intensity values and others with default settings. Segmentation and feature extraction were performed twice at 2-week intervals by a single investigator (KL) who was blinded to the histopathologic results and supervised by a radiologist (KE). Finally, the ComBat harmonization method was applied to the extracted texture features.

### 2.5. Statistical analysis

Regarding conventional CT features, qualitative features were assessed using the Fisher exact test and are expressed as numbers and percentages. Quantitative features were tested for normality using the Shapiro–Wilk test, and the significance of the difference in features between adrenal gland tumor types was examined using one-way analysis of variance. These values are expressed as means and standard deviations for normally distributed values or medians with 95% confidence intervals for non-normally distributed values.

The intraobserver intraclass correlation coefficients (ICC) of the extracted radiomic features were calculated, and all results were >0.90. Therefore, all outcomes derived from the first feature extraction were used. Radiomic features were statistically compared between adrenal gland tumor types using the Kruskal–Wallis H test and are represented as medians and ranges. An ROC curve was constructed, and the area under the ROC curve (AUC) was used to evaluate the diagnostic performance of differentiating AAs, ACCs, and PHEOs using both radiomic and conventional features. AUC values were classified as follows: fail (0.5–0.6), poor (0.6–0.7), fair (0.7–0.8), good (0.8–0.9), or excellent (0.9–1.0). The cut-off value was obtained for features that showed the highest AUC using the maximum Youden index. Diagnostic parameters, including sensitivity, specificity, the positive predictive value (PPV), and the negative predictive value (NPV), were obtained using the cut-off value. The histopathological diagnosis was used as the gold standard of reference for all dogs.

Statistical analyses were performed using MedCalc (version 20.115, MedCalc Software, Ostend, Belgium) and SPSS (version 25.0, IBM Corp., Armonk, NY, USA). For all statistical tests, a *p*-value < 0.05 was considered significant.

## 3. Results

### 3.1. Study population

In total, 40 dogs were collected, and 25 dogs [mean age 10.2 years (range, 8–16 years); 13 spayed females and 12 castrated male dogs] met the inclusion criteria. The following cases were excluded from the study: those with no histopathological results (*n* = 9), benign lesions such as nodular hyperplasia and lipoma (*n* = 1), loss of CT data (*n* = 4), and adrenal mass rupture (*n* = 1). In addition, only 18 of 25 dogs were included in the texture analysis; seven dogs were excluded for the reasons aforementioned. Tumors were diagnosed as AA (*n* = 12, 48%), ACC (*n* = 7, 28%), and PHEO (*n* = 6, 24%), all of which were diagnosed based on surgical excisional biopsy findings. One case of PHEO involved an area with myelolipoma. Two dogs diagnosed with AA were confirmed to have concurrent nodular hyperplasia and extramedullary hematopoiesis, respectively. Capsular invasion was confirmed in only 1 of 6 PHEOs. The breeds were Maltese (*n* = 7), Shih Tzu (*n* = 7), Pomeranian (*n* = 3), Poodle (*n* = 2), Pekingese (*n* = 2), mixed breed (*n* = 2), Yorkshire Terrier (*n* = 1), and Silky Terrier (*n* = 1). Age and sex were not significantly different between the tumor types (Supplementary Table 2).

### 3.2. Conventional CT analysis

#### 3.2.1. Quantitative features

The only significantly associated features were HUmean and HUmax on pre-contrast images (*p* = 0.004 and *p* = 0.03, respectively). Both values were significantly higher in PHEO than in ACC. There were no significant differences between AA and ACC, or between AA and PHEO. The HUmax in the arterial phase was much higher in PHEO than in the other neoplasia, but the difference was not statistically significant (*p* = 0.053). The mean HU difference and maximum diameter were not significantly correlated with the tumor type; however, ACC showed the smallest size among the three tumor types. Comparisons between the tumor types and quantitative CT features are summarized in Supplementary Table 3.

#### 3.2.2. Qualitative features

None of the features were significantly associated with the tumor type (Supplementary Table 4). All cases in the AA group showed smooth tumor margins, but the difference was not statistically significant between the tumor types (*p* = 0.07). No direct vessel invasion (grade 7) or compression (grade 6) was observed in any case. Most of the tumors had a moderate possibility of tumor vascular invasion or adhesion (grades 3, 4, and 5). The majority (AA, 58.3%; ACC, 71.4%; and PHEO, 66.7%) of tumors in the pre-contrast phase and all tumors in the portal and delayed phases showed heterogeneous enhancement. The washout pattern of contrast enhancement was identified only in 1 AA case, and progressive patterns were most frequently observed regardless of the tumor type (*p* = 1.00).

### 3.3. CT texture analysis

The following eight second-order statistics showed significant differences between tumor types on pre-contrast images: the correlation, informational measure of correlation 1 (IMC 1), informational measure of correlation 2 (IMC 2), maximal correlation coefficient (MCC), low gray-level emphasis (LGLE), small dependence low gray-level emphasis (SDLGLE), run entropy, and gray-level non-uniformity normalized (GLNUN) (Supplementary Table 5). No significant features were identified in the delayed phase. All features, except LGLE and SDLGLE, showed significant differences between AA and ACC. ACC and PHEO showed significant differences in all features, except run entropy, IMC 1, and IMC 2. No features showed a significant difference between AA and PHEO. The correlation, MCC, and GLNUN were the most notable features (*p* = 0.013, 0.006, and 0.017, respectively) that showed significant differences between AA and ACC, and between ACC and PHEO.

### 3.4. Evaluation of diagnostic performance

The HUmean and HUmax on the pre-contrast image showed high AUCs for diagnosing ACC and PHEO (Table 1). The cut-off values for differentiating ACC and PHEO are summarized in Supplementary Table 6.

**Table 1:** Areas under the curve of HUmean and HUmax on precontrast images for distinguishing adrenal gland tumor types.

		**AUC value**	**95% CI**
HUmean on precontrast	AA vs. ACC and PHEO	0.532	0.324–0.732
	ACC vs. AA and PHEO	0.865	0.669–0.968
	PHEO vs. AA and ACC	0.860	0.663–0.965
HUmax on precontrast	AA vs. ACC and PHEO	0.538	0.330–0.738
	ACC vs. AA and PHEO	0.806	0.599–0.935
	PHEO vs. AA and ACC	0.785	0.576–0.923

AA, adenoma; ACC, adenocarcinoma; PHEO, pheochromocytoma; AUC, area under the curve; CI, confidence interval; HUmean, mean Hounsfield unit value; HUmax, maximum Hounsfield unit value; vs., versus.

The AUCs, which showed fair (≥0.7) to excellent diagnostic performance in diagnosing each adrenal gland tumor types are summarized in Supplementary Table 7. All radiomic features showed high values (≥0.8) in distinguishing ACC from the other two adrenal gland tumors, and MCC showed the highest AUC (0.969) among them. Run entropy showed the highest AUC (0.787) in discriminating AA, and SLDGLE showed the highest AUC (0.889) in discriminating PHEO. The cut-off values of the radiomic features with the highest AUC are summarized in Table 2.

**Table 2:** Cut-off values of radiomic features with the highest AUC for distinguishing each adrenal gland tumor type.

		**Cut-off value**	**Sensitivity (%)**	**Specificity (%)**
AA vs. ACC and PHEO	Run entropy	>4.116736	80.00	75.00
ACC vs. AA and PHEO	MCC	≤ 0.54275	100.00	92.31
	IMC1	>-0.17065	80.00	100.00
PEHO vs. AA and ACC	SDLGLE	≤ 0.00107	100.00	80.00

AUC, area under the curve; AA, adenoma; ACC, adenocarcinoma; PHEO, pheochromocytoma; MCC, maximum correlation coefficient; IMC2, informational measure of correlation 1; SDLGLE, small dependence low gray-level emphasis; vs., versus.

## 4. Discussion

This study evaluated the feasibility of CT texture analysis for the differentiation of adrenal gland tumors, including cortical AA, ACC, and PHEO, and identified several texture features on pre-contrast images with statistical significance. Only the pre-contrast mean and maximum attenuation values were significant features among the conventional CT characteristics. This study showed that adrenal gland tumors could be distinguished using CT texture analysis, and several texture features represented high sensitivity and specificity for discriminating adrenal gland tumors.

Texture analysis has been used to quantitatively evaluate tumor heterogeneity based on the distribution of gray-level values and the spatial relationship of pixels within an ROI (32). Subtle differences in pixel distribution and intensity can be detected using this image processing technique, which may not be readily identified by the naked eye. This information may reflect the pathology of the tumor, allowing the detection of the lesion and assisting in diagnosis. Therefore, research on texture analysis in the field of imaging diagnosis in human medicine has increased recently.

Eight second-order statistics on pre-contrast images showed significant differences between AA and ACC, and between ACC and PHEO. However, no significant difference was observed between AA and PHEO. ACC was characterized by a lower correlation, MCC, IMC1, IMC2, and run entropy and a higher GLNUN, SDLGLE, and LGLE. The correlation of GLCM is a measure of image linearity, as higher values reflect the high predictability of pixel relationships (33). Several earlier research studies demonstrated that homogeneous or benign lesions express higher values of correlation than malignant lesions (34, 35). MCC quantifies the complexity of the texture, as lower values correspond to a more complex texture of the images. This feature showed the highest AUC in differentiating ACC from the other adrenal gland tumors, and a cut-off value (≤ 0.54275) with high sensitivity and specificity (100 and 92.31%, respectively) was presented. GLNUN of GLSZM quantifies the variability of gray-level values in the images, with a higher value indicating a more variable intensity value. These three features indicate that ACC has more heterogeneous parenchyma with various intensities than the other two adrenal gland tumors. Moreover, these features showed the highest AUC with high sensitivity and specificity at each cut-off value. Thus, the correlation, MCC, and GLNUN would be useful in discriminating ACC using pre-contrast images.

IMC1 and IMC2 are other texture features that indicate the complexity of texture and represent significant differences between ACC and AA. Those with complex and unpredictable textures have values close to 0 (36). ACC showed the lowest value, which was also closest to 0 in both features, indicating a more complex texture.

SDLGLE quantifies the joint distribution of small dependencies with lower gray-level values. A higher value represents lower gray-level values in the ROI with a less homogeneous texture (37). A higher value of LGLE indicates a greater concentration of low gray-level values in the image. Those features showed significantly higher values in ACC than in PHEO, meaning that ACC has more low-intensity pixels in the ROI and a more heterogeneous texture than PHEO.

Although none of the features showed a statistically significant difference between AA and PHEO, PHEO showed lower median SDLGLE values than AA (0.0007 and 0.0013, respectively). This result may be associated with the fact that PHEO showed the highest precontrast attenuation on conventional CT. Additionally, SDLGLE showed the highest AUC (0.889) in diagnosing PHEO, and a cut-off value (≤ 0.00107) was presented, showing high sensitivity and specificity (100% and 80%, respectively). Other features, including MCC and LGLE, also showed a high AUC comparable to that of SDLGLE, which might be useful in distinguishing PHEO from the other adrenal gland tumors.

The greater variability of intensity values with a greater concentration of lower gray-level values in ACC may be consistent with the result that lower pre-contrast mean and maximum HU values are shown in ACC. Statistical significance was identified only between ACC and PHEO, but ACC showed a tendency to express the lowest pre-contrast attenuation value among the three adrenal groups, which is similar to previous studies' findings (8, 9, 38). Only these features showed statistical significance in conventional CT evaluation, also representing a high AUC with high sensitivity and specificity. These features also represented similar cut-off values compared to those in previous studies, which showed values of 48 HU for PHEO and 39 HU for ACC (9). Although statistical significance was not verified, texture features herein showed a comparable or higher AUC in discriminating ACC and PHEO, especially in differentiating ACC. Therefore, this finding may suggest the feasibility of CT texture analysis in canine adrenal glands using pre-contrast imaging.

It is meaningful to be able to differentiate adrenal gland tumors, especially ACC, in pre-contrast images through texture analysis. First, post-contrast images are affected by various factors, including the contrast agent injection rate and concentration, scan delay time, and scan parameters. However, since the texture features extracted from the pre-contrast images are more dependent on the characteristics of the tumor itself, objective evaluation is possible (39). Second, texture analysis may be useful as an ancillary diagnostic tool for adrenal incidentaloma in cases without post-contrast images.

Several studies that investigated CT texture analysis in human adrenal gland lesions reported that ACC and metastasis of other neoplasias showed more heterogeneity than AA (40, 41). Another study reported that lipid-poor AA showed a more homogeneous and finer texture than non-AA, which includes PEHO, ACC, and metastasis (42). Conversely, previous research has shown more heterogeneity in AA than in ACC, which showed a smaller diameter than AA (43). The smaller size of the malignant lesions may reflect the early stage of cancer without typical angiogenesis or necrosis. Therefore, even malignant lesions can exhibit homogeneous, uniform, and fine textures comparable to those of benign lesions. A similar result was identified in another study in which adrenal metastatic lesions smaller than benign lesions showed a more homogeneous and fine texture (44). Similarly, AA had a larger diameter in both the short and long axes than ACC in this study, although this was not statistically significant. Additionally, AA showed a higher value of run entropy than ACC. Run entropy is correlated with randomness in the distribution of run lengths and gray levels, with higher values indicating a more heterogeneous texture. This finding is contrary to the result mentioned earlier, but an overlap of values was observed between the three adrenal gland tumor groups. Moreover, other significant features in this study demonstrated a more heterogeneous and complex texture of ACC. Thus, further evaluation with a larger number of patients is needed to validate this result.

Texture features with significant differences were identified on post-contrast images in previous human studies (39–41, 43, 44), but none of the features were significantly different between adrenal gland tumor types in the delayed phase of this study. This result could be related to the heterogeneity of AA identified on the pre-contrast images. According to the histopathological examination, the following characteristics are highly correlated with canine ACC: a larger size (>2 cm), fibrosis, capsular invasion, trabecular growth pattern, decreased cytoplasmic vacuolation, hemorrhage, necrosis, and increased proliferation index (Ki67) (45). However, AA and ACC may show overlapping histopathological characteristics; thus, differentiating these tumors is challenging (46). ACC also exhibits various tumor cell growth patterns, including trabecular, lobular, and nest-like patterns (46). As mentioned previously, the average tumor diameter of AA exceeded 2 cm, representing a larger tumor size than that of ACC. In a previous study, adrenal gland tumors could not be effectively distinguished using the maximum and minimum HU values and parenchymal heterogeneity in the delayed phase (8). Another study showed no difference between the degree of contrast enhancement or HU difference and the type of adrenal gland tumor (7). This study emphasized that the tumor size itself has a greater influence on parenchymal necrosis, hemorrhage, and tumor heterogeneity than does tumor malignancy. In addition, although the ComBat harmonization technique was used to normalize the features, the scanners and scanning protocols, including the injection rate of the contrast agent and scan delay time, were diverse in a small number of dogs. Thus, further studies are needed to investigate the differences in radiomic features between adrenal gland tumor types on post-contrast images under the same conditions.

The present study has several limitations. First, the small number of dogs included in this study may have limited the power of the statistical analysis. Because of the retrospective nature of this study, only patients with a final diagnosis based on a histological examination after adrenalectomy were selected. This may have caused selection bias because a tentative diagnosis is often made without a histological diagnosis based on evident clinical signs or biochemical examination results. Second, the patients' clinical information, including clinical symptoms related to adrenal gland tumors and hormone secretion levels, were not included in this study. As reported in a previous human study that demonstrated the feasibility of differentiating functional and non-functional adrenal incidentalomas by CT texture analysis, different results could be obtained depending on whether the adrenal gland tumor is functional (47, 48). Further studies on the differences in texture features between functional and non-functional adrenal masses would have clinical value. Third, all 93 radiomic features were included in the feature selection herein. Feature reduction is an important step to avoid data overfitting, which could have also limited the power of the statistical analysis. Various statistical methods are used in radiomic studies, including intraclass correlation coefficient analysis, univariate analysis, multivariate analysis, and machine learning. Many radiomic studies tested the reproducibility of segmentation and feature extraction between multiple inspections by a single observer and between different observers by the intraclass correlation coefficient (40, 48–51). Although a high intraclass correlation coefficient (≥0.9) was confirmed for all texture features in the present study, non-reproducible features may have been identified if interobserver agreement analysis had been performed, and several features might have been excluded. However, feature reduction can also result in the elimination of informative features; hence, carefully choosing feature reduction and selection methods that are most suitable for the study is important. Lastly, model establishment and cross-validation using training and validation sets were impossible owing to the small sample size. A statistical model that aims to predict tumor characteristics, such as benign vs. malignant, prognosis, including recurrence or response to treatment, and mean survival time, needs to be built. The performance and generalizability of a model are determined by its validation in a new test set. Although this process was not conducted, the diagnostic performance of the features was evaluated using an ROC curve, which achieved a high AUC value herein. ROC curve analysis is suitable for evaluation of the diagnostic performance of a feature when univariate analysis is conducted, and such a model can be used as a benchmark for multivariate analysis (15). Finally, various CT scanners and imaging protocols were selected, and the ComBat harmonization technique was used to compensate for this variability. Although the minimum number of patients required to apply this technique has not been fully investigated, significant features were identified using it, and meaningful results were obtained.

In conclusion, this study demonstrates that evaluation of canine primary adrenal gland tumors using CT texture analysis may be useful for distinguishing adrenal gland tumor types and assisting in clinical decision-making. Statistical model establishment and cross-validation in further prospective studies with larger populations are warranted to verify these results.

## Data availability statement

The original contributions presented in the study are included in the article/Supplementary material, further inquiries can be directed to the corresponding author.

## Ethics statement

The animal studies were approved by Institutional Animal Care and Use Committee. The studies were conducted in accordance with the local legislation and institutional requirements. Written informed consent was obtained from the owners for the participation of their animals in this study.

## Author contributions

KL contributed to the study design, data collection, data interpretation, and manuscript writing. JG contributed to the data interpretation. JJ, JH, and JKw contributed to the data acquisition and collection. JKi and KE contributed to the study design and manuscript editing. All authors reviewed and approved the final submitted manuscript.
