# Supplementary material for: Feasibility study of computed tomography texture analysis for evaluation of canine primary adrenal gland tumors
Source: Front Vet Sci. 2023 Aug 30;10:1126165. doi: 10.3389/fvets.2023.1126165 (PMC10499047; doi:10.3389/fvets.2023.1126165)
Supplement: Supplementary file 1 [file Data_Sheet_1.pdf]

Supplementary Material

Feasibility Study of Computed Tomography Texture Analysis for Evaluation of Canine Primary Adrenal Tumors

Kyungsoo Lee, Jinhyung Goh, Jaeyoung Jang, Jeongyeon Hwang, Jungmin Kwak, Jaehwan Kim, Kidong Eom*

*** Correspondence:** eomkd@konkuk.ac.kr

1 Supplementary Tables

Supplementary Table 1. Computed tomography scanning parameters

	CT scanner	Tube voltage (kVp)	mAs	Slice thickness (mm)
1	General Electric Brivo CT385 series	120	69	1.25
2	General Electric Lightspeed plus	120	200	1.30
3	General Electric Revolution ACT	120	85	2.50
4	Toshiba Aquilion	120	250	2.0
5	Toshiba Aquilion Lightning	120	150	1.0
		120	180 [a], 150 [b]	1.0
6	Siemens Somatom go.Now	130	113 [a], 125 [b]	2.0 [a], 1.0 [b]
		130	111	1.5
7	Siemens Emotion 16	130	147 [a], 140 [c], 129 [b]	1.0
8	Siemens Somatom Scope	130	147 [a], 165 [b]	2.0
9	Philips Access CT	120	150	1.0

[a] Precontrast, [b] delayed phase, [c] arterial and portal phases.
CT, computed tomography.

Supplementary Table 2. Age and sex of the dogs

		AA (n = 12)	ACC (n = 7)	PHEO (n = 6)	*p*-value
Age (years)		10.6 ± 2.3	10.0 ±1.7	9.7 ± 1.0	0.62
Sex	Castrated male	5 (41.7%)	3 (42.8%)	4 (66.7%)	0.67
	Spayed female	7 (58.3%)	4 (57.2%)	2 (33.3%)	

Ages are presented as mean ± standard deviation.
A *p*-value <0.05 was considered statistically significant.
AA, adenoma; ACC, adenocarcinoma; PHEO, pheochromocytoma.

Supplementary Table 3. Quantitative computed tomography features for distinguishing the adrenal tumor types

		AA (n = 12)	ACC (n = 7)	PHEO (n = 6)	*p*-value
Maximal diameter in the short axis (mm)		20.85 ± 6.07	16.65 ± 2.89	23.57 ± 7.82	0.51
Maximal diameter in the long axis (mm)		26.48 ± 5.14	23.67 ± 6.42	28.44 ± 5.17	0.83
HUmean	Precontrast	37.91 ± 8.41	29.51 ± 5.57 [a]	46.43 ± 7.30 [a]	0.004
	Arterial phase	50.85 ± 20.15	60.54 ± 17.43	66.34 ± 23.26	0.52
	Portal phase	75.97 ± 43.21	105.87 ± 54.26	87.70 ± 48.05	0.54
	Delayed phase	101.92 ± 45.13	108.77 ± 50.37	119.03 ± 39.01	0.75

All data are presented as mean ± standard deviation.
A *p*-value <0.05 was considered statistically significant. [a] There is a significant difference between the two tumor types.
AA, adenoma; ACC, adenocarcinoma; PHEO, pheochromocytoma; HUmean, mean Hounsfield unit value

Supplementary Table 3. Quantitative computed tomography features for distinguishing the adrenal tumor types (continued)

		AA (n = 12)	ACC (n = 7)	PHEO (n = 6)	***p*-value**
HUmax	Precontrast	62.50 ± 12.07	52.87 ± 10.85 [a]	71.33 ± 11.57 [a]	0.03
	Arterial phase	122.50 ± 13.05	141.80 ± 64.85	249.50 ± 31.81	0.053
	Portal phase	164.44 ± 72.03	198.20 ± 86.07	251.50 ± 91.21	0.36
	Delayed phase	166.50 ± 53.17	171.00 ± 53.89	186.33 ± 50.66	0.64
HUmin	Precontrast	14.66 ± 10.86	6.42 ± 6.24	17.66 ± 10.42	0.16
	Arterial phase	10.00 ± 14.04	15.60 ± 12.17	30.00 ± 15.55	0.21
	Portal phase	23.55 ± 19.15	28.80 ± 19.03	34.00 ± 19.42	0.77
	Delayed phase	38.41 ± 42.90	51.00 ± 43.79	57.50 ± 30.15	0.76

All data are presented as mean ± standard deviation.
A *p*-value <0.05 was considered statistically significant. [a] There is a significant difference between the two tumor types.
AA, adenoma; ACC, adenocarcinoma; PHEO, pheochromocytoma; HUmax, maximum Hounsfield unit value; HUmin, minimum Hounsfield unit value.

Supplementary Table 3. Quantitative computed tomography features for distinguishing the adrenal tumor types (continued)

		AA (n = 12)	ACC (n = 7)	PHEO (n = 6)	***p*-value**
Mean HU difference	Arterial phase	14.97 ± 17.30	29.26 ± 21.53	18.12 ± 22.59	0.45
	Portal phase	43.69 ± 29.85	76.71 ± 60.56	39.48 ± 47.38	0.37
	Delayed phase	64.01 ± 41.47	79.26 ± 55.51	72.60 ± 35.98	0.76

All data are presented as mean ± standard deviation.
A *p*-value <0.05 was considered statistically significant.
AA, adenoma; ACC, adenocarcinoma; PHEO, pheochromocytoma; HU, Hounsfield unit.

Supplementary Table 4. Qualitative computed tomography features for distinguishing the adrenal tumor types

		AA (n = 12)	ACC (n = 7)	PHEO (n = 6)	*p*-value
Tumor location	Right	7/12 (58.3%)	3/7 (42.8%)	4/6 (66.7%)	0.76
	Left	5/12 (42.7%)	4/7 (57.2%)	2/6 (33.3%)	
Tumor shape	Round	2/12 (16.7%)	-	-	0.56
	Oval	1/12 (8.3%)	1/7 (14.3%)	2/6 (33.3%)	
	Lobulated	9/12 (75.0%)	6/7 (85.7%)	4/6 (66.7%)	
Tumor contour	Smooth	12/12 (100%)	1/7 (14.3%)	4/6 (66.7%)	0.07
	Irregular	-	6/7 (85.7%)	2/6 (33.3%)	
Intratumoral calcification	Absent	11/12(91.6%)	5/7 (71.4%)	4/6 (66.7%)	0.45
	Present	1/12 (8.4%)	2/7 (28.6%)	2/6 (33.3%)	

A *p*-value <0.05 was considered statistically significant.
AA, adenoma; ACC, adenocarcinoma; PHEO, pheochromocytoma.

Supplementary Table 4. Qualitative computed tomography features for distinguishing the adrenal tumor types (continued)

		AA (n = 12)	ACC (n=7)	PHEO (n = 6)	*p*-value
Possibility of adhesion or invasion of the adjacent vessel	Weak or no contact	4/12 (33.3%)	4/7 (57.2%)	1/6 (16.7%)	0.32
	Moderate	8/12 (66.7%)	3/7 (42.8%)	5/6 (83.3%)	
	Strong	-	-	-	
Type of CE at precontrast	Homogeneous	5/12 (41.7%)	2/7 (28.6%)	2/6 (33.3%)	0.87
	Heterogeneous	7/12 (58.3%)	5/7 (71.4%)	4/6 (66.7%)	
Type of CE in the arterial phase	Homogeneous	-	-	-	-
	Heterogeneous	8/8 (100%)	4/4 (100%)	2/2 (100%)	
Type of CE in the portal phase	Homogeneous	-	-	-	-
	Heterogeneous	8/8 (100%)	4/4 (100%)	5/5 (100%)	
Type of CE in the delayed phase	Homogeneous	-	-	-	-
	Heterogeneous	12/12 (100%)	7/7 (100%)	6/6 (100%)	
Rim enhancement	Absent	8/12 (66.7%)	3/7 (42.8%)	5/6 (83.3%)	0.32
	Present	4/12 (33.3%)	4/7 (57.2%)	1/6 (16.7%)	

A *p*-value <0.05 is considered statistically significant.
AA, adenoma; ACC, adenocarcinoma; PHEO, pheochromocytoma; CE, contrast enhancement.

Supplementary Table 4. Qualitative computed tomography features for distinguishing the adrenal tumor types (continued)

		AA (n = 12)	ACC (n = 7)	PHEO (n = 6)	*p*-value
Pattern of CE	Stable	4/12 (33.3%)	2/7 (28.6%)	-	1.00
	Progressive	7/12 (58.3%)	5/7 (71.4%)	2/2 (100%)	
	Washout	1/12 (8.4%)	-	-	
Degree of CE in the arterial phase	Minimal	8/8 (100%)	4/5 (80.0%)	2/2 (100%)	0.46
	Moderate	-	1/5 (20.0%)	-	
	Intense	-	-	-	
Degree of CE in the portal phase	Minimal	4/9 (44.4%)	2/5 (40.0%)	1/2 (50.0%)	0.51
	Moderate	5/9 (45.6%)	2/5 (40.0%)	1/2 (50.0%)	
	Intense	-	1/5 (20%)	-	
Degree of CE in the delayed phase	Minimal	3/12 (25.0%)	1/7 (14.3%)	1/6 (16.7%)	0.46
	Moderate	7/12 (58.3%)	5/7 (71.4%)	4/6 (6.6%)	
	Intense	2/12 (16.7%)	1/7 (14.3%)	1/6 (16.7%)	

A *p*-value <0.05 was considered statistically significant.
AA, adenoma; ACC, adenocarcinoma; PHEO, pheochromocytoma; CE, contrast enhancement.

Supplementary Table 5. Radiomic features with a significant difference between the adrenal tumor types on precontrast images

Radiomic feature		AA (n = 10)	ACC (n = 5)	PHEO (n = 3)	*p*-value
GLCM	Correlation	0.53 [0.45, 0.61] [a*]	0.41 [0.39, 0.52] [a*, b]	0.59 [0.53, 0.60] [b]	0.013
	IMC 1	-0.20 [-0.24, -0.17] [a*]	-0.15 [-0.18, -0.13] [a*]	-0.22 [-0.23, -0.17]	0.014
	IMC 2	0.60 [0.47, 0.67] [a]	0.49 [0.44, 0.57] [a]	0.64 [0.54, 0.65]	0.036
	MCC	0.59 [0.49, 0.66] [a*]	0.48 [0.44, 0.54] [a*, b]	0.63 [0.61, 0.65] [b]	0.006
GLDM	LGLE	0.02 [0.02, 0.05]	0.05 [0.03, 0.05] [b]	0.02 [0.02, 0.02] [b]	0.038
	SDLGLE	0.0013 [0.0005, 0.0023]	0.0018 [0.0012, 0.0026] [b]	0.0007 [0.0006, 0.0011] [b]	0.032
GLRLM	Run entropy	4.22 [3.99, 4.44] [a]	4.03 [3.92, 4.12] [a]	4.20 [4.08, 4.27]	0.031
GLSZM	GLNUN	0.18 [0.17, 0.25] [a*]	0.22 [0.21, 0.26] [a*, b]	0.18 [0.16, 0.20] [b]	0.017

All data are presented as median and range (minimum, maximum)
A *p*-value <0.05 was considered statistically significant. * $p<0.01$. [a, b] The same superscript letter indicates a significant difference between the two tumor types.
AA, adenoma; ACC, adenocarcinoma; PHEO, pheochromocytoma; GLCM, gray level co-occurrence matrix; GLDM, gray-level dependence matrix; GLRLM, gray-level run length matrix; GLSZM, gray-level size zone matrix; IMC1, informational measure of correlation 1; IMC2,

informational measure of correlation 2; MCC, maximum correlation coefficient; LGLE, low gray-level emphasis; SDLGLE, small dependence low gray-level emphasis; GLNUN, gray-level non-uniformity normalized.

Supplementary Table 6. The cut-off value of HUmean and HUmax on precontrast images for distinguishing adrenal tumor types

		Cut-off value	Sensitivity (%)	Specificity (%)
HUmean on precontrast	ACC vs. AA and PHEO	≤ 32.47	85.71	77.78
	PHEO vs. AA and ACC	> 45.21	83.33	94.74
HUmax on precontrast	ACC vs. AA and PHEO	≤ 63	100	61.11
	PHEO vs. AA and ACC	> 71	66.67	94.74

AA, adenoma; ACC, adenocarcinoma; PHEO, pheochromocytoma; HUmean, mean Hounsfield unit value; HUmax, maximum Hounsfield unit value; vs., versus.

Supplementary Table 7. The area under the curve (≥ 0.7) of radiomic features for distinguishing adrenal tumor types on precontrast images

			AUC value	95% CI
AA vs. ACC and PHEO	GLCM	IMC1	0.762	0.507–0.927
	GLRLM	Run entropy	0.787	0.566–1
	GLSZM	GLNUN	0.725	0.452–0.997
ACC vs. AA and PHEO	GLCM	Correlation	0.938	0.719–0.998
		IMC 1	0.954	0.741–0.999
		IMC 2	0.892	0.657–0.987
		MCC	0.969	0.764–0.997
	GLDM	LGLE	0.846	0.601–0.970
		SDLGLE	0.831	0.653–0.986
	GLRLM	Run entropy	0.908	0.677–0.998
	GLSZM	GLNUN	0.938	0.719–0.998

AA, adenoma; ACC, adenocarcinoma; PHEO, pheochromocytoma; AUC, area under the curve; CI, confidence interval; GLCM, gray-level co-occurrence matrix; GLDM, gray-level dependence matrix; GLRLM, gray-level run length matrix; GLSZM, gray-level size-zone matrix; IMC1, informational measure of correlation 1; IMC2, informational measure of correlation 2; MCC, maximum correlation coefficient; LGLE, low gray-level emphasis; SDLGLE, small dependence low gray-level emphasis; GLNUN, gray-level non-uniformity normalized; vs., versus.

Supplementary Table 7. The area under the curve (≥ 0.7) of radiomic features for distinguishing adrenal tumor types on precontrast images (continued)

			AUC value	95% CI
PHEO vs. AA and ACC	GLCM	IMC2	0.733	0.477–0.91
		Correlation	0.800	0.575–0.948
		MCC	0.844	0.599–0.969
	GLDM	LGLE	0.844	0.599–0.969
		SDLGLE	0.889	0.653–0.986
	GLSZM	GLNUN	0.733	0.477–0.91

AA, adenoma; ACC, adenocarcinoma; PHEO, pheochromocytoma; AUC, area under the curve; CI, confidence interval; GLCM, gray-level co-occurrence matrix; GLDM, gray-level dependence matrix; GLSZM, gray-level size-zone matrix; IMC2, informational measure of correlation 2; MCC, maximum correlation coefficient; LGLE, low gray-level emphasis; SDLGLE, small dependence low gray-level emphasis; GLNUN, gray-level non-uniformity normalized; vs., versus
